# Pulmonary delivery of siRNA lipoplexes and lipid nanoparticles using a vibrating mesh nebuliser

**DOI:** 10.1016/j.ejps.2025.107386

**Published:** 2026-01-01

**Authors:** Michael T. Neary, Lianne M. Mulder, Ciaran O. Leime, Ronan MacLoughlin, Brunella Grassiri, Łukasz Baranowski, Piotr S. Kowalski, Abina M. Crean, Katie B. Ryan

**Affiliations:** a SSPC, the SFI Research Centre for Pharmaceuticals, School of Pharmacy, University College Cork, Ireland; b School of Pharmacy, University College Cork, Ireland; c Aerogen Ltd. Galway Business Park, Galway, Ireland; d School of Pharmacy and Pharmaceutical Sciences, Panoz Institute, Trinity College Dublin, Dublin 2, Ireland; e Preclinical Drug Development Facility at IN-MOL-CELL, International Institute of Molecular and Cell Biology, Trojdena 4, 02-109 Warsaw, Poland; f APC Microbiome, University College Cork, Ireland

**Keywords:** Nebulisation, siRNA, Pulmonary delivery, Nanocarriers, Pegylation, Droplet size

## Abstract

Inhalation via nebulisation is a promising method to deliver high concentrations of siRNA to the lung epithelium in a direct and non-invasive manner for the treatment of numerous respiratory-related illnesses. However, nebulisation can be destructive towards siRNA nanocarriers leading to loss of siRNA and a diminished therapeutic outcome. Herein, we sought to explore how the nebulisation process, including adjustments in aerosol droplet size impacts the physicochemical properties of several lipid-based siRNA nanocarrier formulations. These included PEGylated and non-PEGylated cationic DOTAP-based lipoplexes (LPXs) and C12–200 based lipid nanoparticles (LNPs). Two Aerogen® Pro vibrating mesh nebuliser devices with capacities to generate aerosols of differing volumetric mean diameters (VMD) were utilised. The aerosol droplet sizes for the different siRNA formulations were 4.80 to 4.89 µm (High VMD device) and 3.56 to 3.59 µm (Low VMD device) demonstrating that the emitted droplet size distribution was consistent across multiple siRNA nanocarrier types. Further, the formulations exhibited mass median aerodynamic diameters (MMAD) of 4.03 – 4.84 µm (High VMD device) indicating their potential for targeting siRNA lung deposition. Aggregation in both lipoplex formulations and a significant reduction in LNPs’ siRNA encapsulation efficiency were observed. *In vitro* studies in Firefly luciferase (Fluc) expressing alveolar A549 cells demonstrated that cell viability and Fluc knockdown were generally unaffected by nebulisation. However, Fluc knockdown varied depending on formulation type and was highest for LNPs (93 %) and lowest for the PEGylated LPXs (max 30 %). Overall, this study shows that aerosols with consistent droplet size can be generated but the choice of nanocarrier impacts the stability and delivery efficacy and requires careful consideration for efficient nebulised siRNA delivery.

## Graphical abstract

**Figure:**
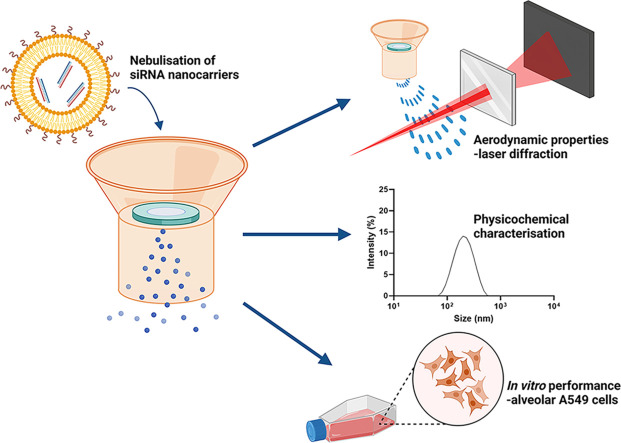

## 1 Introduction

Small interfering RNA (siRNA) offers enormous therapeutic potential owing to its ability to inhibit gene expression via RNA interference (RNAi). Upon cell entry, siRNA can bind to a target mRNA with a complementary nucleotide sequence culminating in the mRNA’s cleavage and inhibition of its translation into protein *i.e.*, gene silencing (Hammond et al., 2021). The lungs are one of many organs that have been identified as a target for siRNA treatment. Several preclinical studies have demonstrated the promise of siRNA for treating a diverse range of respiratory conditions such as lung cancer (Conte et al., 2021), asthma (Keil et al., 2020), COPD (Dong et al., 2019) and infectious diseases for example, influenza (Blanchard et al., 2021). Pulmonary administration via inhalation represents a direct, convenient, and non-invasive means of delivering high quantities siRNA to target sites in the lungs with minimal systemic side effects (Chow et al., 2020; Verma et al., 2013). Aerosolization by nebulisation of aqueous solutions/suspensions producing respirable droplets, is an effective means of delivering large drug doses during the patients’ tidal breathing (Martin and Finlay, 2015). Vibrating mesh nebulisers have emerged in the market more recently than traditional jet and ultrasonic nebulisers (Gaspar et al., 2010) and have become the preferred device type due to their efficiency in delivering drug to the lung, low residual volume and discreet and compact nature (Barjaktarevic and Milstone, 2020).

Despite its therapeutic potential for local lung conditions, the nebulised administration of siRNA presents several obstacles. Some previous studies have shown that the shear forces of nebulisation can reduce the integrity of naked siRNA (Sharma et al., 2013; Tang et al., 2020), although siRNA is generally considered more robust in this regard compared to the more delicate mRNA. Naked siRNA also encounters many extracellular barriers *e.g.* mucus penetration (Almeida et al., 2019), while its physicochemical properties; negative charge, size, and hydrophilicity impede its uptake across the cell membrane and access to the intracellular target (Kauffman et al., 2016). To address these challenges, siRNA has been incorporated into a range of nanosized vectors for nebulised delivery including liposomes (Guan et al., 2021), lipid nanoparticles (LNPs) (Kim et al., 2020; Zhang et al., 2020), polymeric based systems (Hibbitts et al., 2020) as well as lipid-polymer hybrid-based systems (D’Angelo et al., 2018). LNPs, approved in mRNA vaccines targeting the COVID-19 virus, are amongst the most efficient non-viral delivery vectors for RNA (Nele et al., 2024). Further, they have shown potential for nebulised administration (Kim et al., 2022; Lokugamage et al., 2021). However, LNPs can be destabilised when exposed to nebulisation. For example, Zhang et al., noted a size increase post nebulisation in all 18 of their mRNA LNP formulations based on dilinoleylmethyl-4-dimethylaminobutyrate (Dlin-MC3-DMA), which were formulated with a diverse range of structural lipids, PEG lipids and molar % compositions (Zhang et al., 2020). Formulation optimisation to maximise LNP stabilisation during the nebulisation process is a key consideration and in particular, PEG is shown to be highly influential (Lokugamage et al., 2021). Kim et al. for instance, were able to enhance LNP stability during nebulisation by increasing the amount of PEG in their LNP formulation (Kim et al., 2022). Lipoplexes formed between RNA and cationic liposomes containing lipids such as 1,2-dioleoyl-3-trimethylammonium-propane (DOTAP) have also been used for nebulised RNA delivery (Guan et al., 2021; McCarthy et al., 2023; Mainelis et al., 2013). Their cationic charge promotes interaction with the cell and endosomal membranes facilitating cell entry (Liu et al., 2020), however, similar to LNPs, lipoplexes can become unstable during nebulisation (Guan et al., 2021). Whether PEGylation can also enhance lipoplex stability as previously demonstrated for LNPs (Kim et al., 2022) has not been reported to date.

The performance of a nebulised siRNA delivery system depends not only on the type of nanocarrier formulation employed but also on the nebulisation process itself (Neary et al., 2024). To elicit a therapeutic effect, nebulisers must produce aerosol droplets of an appropriate size range for inhalation (1–5 µm for peripheral lung deposition) (Mangal et al., 2017). To date however, there is a lack of studies investigating the interplay between a nebuliser’s aerodynamic properties and the characteristics of the siRNA nanocarrier being nebulised. In a study by Kim et al., mRNA LNPs were nebulised with two Aeroneb® Lab vibrating mesh nebulisers of different droplet size specifications (4–6 µm and 2.5–4 µm) and observed a similar increase in nanoparticle size from around 70–90 nm to around 170 nm for both units. However, neither droplet size distribution nor mRNA encapsulation efficiency (EE %) were measured alongside LNP size (Kim et al., 2020). Miao et al. assessed the impact of aerosolization technique on the physical properties of mRNA LNPs across several device types including ultrasonic, jet and vibrating mesh nebulisers and a soft mist inhaler. However, their study focused primarily on the influence of differences in atomisation energy rather than examining the interplay between emitted droplet size and LNP formulations (Miao et al., 2023). It remains unclear whether the aerosol droplet size distribution varies with a change from one siRNA formulation type to another. Further, the impact of altering the emitted droplet size, and the consequences on the physicochemical stability across different nebulised siRNA nanocarriers has not been properly investigated. Ideally, siRNA nanocarriers should maintain stability post nebulisation characterised by a uniform particle size with no aggregation, low PDI and high siRNA EE %. A higher surface to volume ratio due to a reduced droplet size (Ji et al., 2012) leads to greater exposure of nanocarriers to air-liquid interfacial forces, which are known to destabilise LNPs (Matthessen et al., 2024). It is conceivable therefore that a nebuliser emitting aerosol with a smaller volumetric mean diameter (VMD) could cause greater physical instability of siRNA nanocarriers. This could have implications for nanocarrier size, polydispersity, siRNA loading and EE %.

The first aim of this study was to compare the aerosol properties of a range of lipid-based siRNA nanocarriers nebulised with two Aerogen® Pro vibrating mesh nebulisers capable of generating differing droplet size distributions *i.e.*, a High resultant VMD and Low VMD. Aerosol properties were evaluated using laser diffraction (both devices) and cascade impaction (High VMD device only). Several lipid-based formulations were compared including DOTAP-based lipoplexes (non-PEG LPXs) and PEGylated lipoplexes containing DOTAP (PEG LPXs). LNPs containing the benchmark ionizable, lipidoid, 1,1-((2-(4-(2-((2-(bis(2-hydroxydodecyl)amino)ethyl)(2-hydroxydodecyl) amino)ethyl)piperazin-1-yl)ethyl)azanediyl)bis(dodecan-2-ol) (C12–200) were also investigated. C12–200 LNPs have been shown as efficient delivery vectors of RNA via the pulmonary route (Jiang et al., 2023; Ongun et al., 2024). Secondly, we sought to investigate if changes in aerosol characteristics would affect the physicochemical stability of our chosen nanocarriers that were loaded with Firefly luciferase (Fluc) siRNA. Nanocarrier size and siRNA EE % were measured pre and post nebulisation to assess any impact on physicochemical stability. Lastly, we aimed to compare the impact of nebulisation on siRNA nanocarrier *in vitro* performance and to thus gauge their suitability for nebulised pulmonary siRNA delivery. Fluc knockdown levels and cell viability were measured pre and post nebulisation in alveolar epithelium derived, A549 cells to evaluate transfection efficiency and cytotoxicity in a lung relevant *in vitro* model (Bhowmick and Gappa-Fahlenkamp, 2016).

## 2 Materials and methods

### 2.1 Materials

Custom designed siRNA duplexes (21 nucleotides in length) targeting the firefly luciferase gene (luc2) (sense CGACAAGCCUGGCGCAGUAUU; antisense UACUGCGCCAGGCUUGUCGUU), Cy5-labeled siRNA (sense Cy5-CGACAAGCCUGGCGCAGUAUU; antisense UACUGCGCCAGGCUUGUCGUU) and non-targeting siRNA (sense GAUUAUGUCCGGUUAUGUAUU; antisense UACAUAACCGGACAUAAUCUU) were purchased from Horizon Discovery Biosciences Ltd (Cambridge, UK). 1,1-((2-(4-(2-((2-(bis(2-hydroxydodecyl)amino)ethyl)(2-hydroxydodecyl) amino)ethyl)piperazin-1-yl)ethyl)azanediyl)bis(dodecan-2-ol) (C12–200) was purchased from Corden Pharma (Liestal, Switzerland). 1,2-dioleoyl-3-trimethylammonium-propane (DOTAP), cholesterol, N-(Methylpolyoxyethylene oxycarbonyl)-1,2-dimyristoyl-sn‑glycero-3-phosphoethanolamine (DMPE-PEG_2000_) and 1,2-distearoyl-sn‑glycero-3-phosphocholine (DPSC) were sourced from Avanti Polar Lipids (Alabaster, AL, USA). Unless otherwise stated, all other chemicals and materials were purchased from Merck, Ireland. All plasticware used, unless otherwise stated, was purchased from Sarstedt (Wexford, Ireland).

### 2.2 Preparation of siRNA formulations using microfluidic technology

siRNA formulations were manufactured using a microfluidic staggered herringbone mixer design chip and Harvard Apparatus Pump 33 Dual Drive System (Havard Apparatus, MA, USA). An aqueous phase containing siRNA in either 1X phosphate-buffered saline (PBS) (pH 7.4) or 10 mM sodium citrate buffer (pH 3.0) (both purchased from Thermo Fisher Scientific, Dublin, Ireland) was mixed with an organic phase containing the required lipid components in ethanol, Table 1. Prior to microfluidic mixing, the organic phase was prepared by combining the appropriate volumes of each individual lipid dissolved in ethanol. The mass ratio of C12–200: siRNA in the LNPs was 5:1. For the lipoplex formulations (non-PEG LPXs and PEG LPXs) an N/P ratio of 5:1 was used *i.e.*, the ratio of positively charged amine groups in the cationic lipid to the negatively charged phosphate groups present in siRNA. A flow rate ratio (FRR) (aqueous: organic) of 5:1 was used for all formulations. Preliminary work also explored a FRR of 3:1 which produced comparable results to 5:1 in terms of nanocarrier size, PDI and EE % (**Fig S1**). After preparation, all formulations were immediately dialyzed in 1X PBS using 10 K MWCO Slide-A-Lyzer dialysis devices (Thermo Fisher Scientific, Dublin, Ireland) for 4 h to remove residual ethanol. The dialysate was replaced with fresh PBS after 2 h.

**Table 1:** siRNA formulations produced using microfluidic technology including details of organic phase and aqueous buffer used.

Formulation	Organic phase components and molar % ratio	Aqueous buffer
non-PEG LPXs	DOTAP: Cholesterol (50:50)	1X PBS
PEG LPXs	DOTAP: Cholesterol: DMPE-PEG_2000_ (50:45:5)	1X PBS
LNPs	C12–200: Cholesterol: DPSC: DMPE-PEG_2000_ (50:38.5:10:1.5)	10 mM sodium citrate

### 2.3 Nebulisation of siRNA nanocarriers

Two Aerogen® Pro nebulisers with capacity to generate different aerosol size distributions were used: Aerogen® Pro nebuliser, in combination with the Aerogen® USB controller, Aerogen, Galway, Ireland. These were designated as “Low VMD device” and the higher droplet size distribution, “High VMD device”. Both devices were used to nebulise each siRNA formulation, and the aerosol was characterised using laser diffraction analysis. Nanocarrier size and siRNA EE % were also assessed. Cascade impaction analysis, zeta potential measurement, gel electrophoresis and *in vitro* A549 cell studies were conducted on samples nebulised using the High VMD device only. Nebulisation involved pipetting the samples (≥ 100 µL) directly into the nebuliser reservoir. The nebulised droplets were collected in a 50 mL Falcon tube which was secured to the nebuliser outlet with Parafilm® and submerged in ice. The nebuliser was operated until aerosol was no longer produced. For cell culture experiments, the nebuliser was sterilised beforehand in an autoclave at 121 °C for a minimum of 15 min.

### 2.4 Nebulised droplet size characterization using laser diffraction

Aerosol droplet size distributions described by Volumetric Mean Diameter (VMD) (Dv50) were measured using a Malvern Spraytec droplet size analyzer (Malvern Instruments Ltd., UK) with RT Sizer software as previously described (MacLoughlin et al., 2009). Briefly, the Aerogen® Pro nebuliser was connected to the inhalation port of the Spraytec using a bespoke connector, and a 5 L/min vacuum flow was implemented through the system, ensuring complete entrainment of the aerosol as it exited the nebuliser. This vacuum ensured a laminar flow and thus a reduction in droplet size growth due to turbulent collision with other droplets. Additionally, the flow rate helped ensure a single pass through the laser for each droplet. Prior to testing, the Spraytec system was calibrated using a dedicated calibration system available from the manufacturer. Droplet size was characterised using 100 µL volumes for each of the different formulations. PBS devoid of any nanocarriers was also nebulised as a control as this was the liquid medium in which all nanocarriers were suspended. The aerosol flow rate (mL/min) was recorded concurrently for each formulation as an estimate of the rate of aerosol emission.

### 2.5 Cascade impaction using a next generation impactor (NGI)

*In vitro* deposition of nebulised siRNA nanocarriers was determined using an NGI (Copley Scientific, Nottingham, UK). The Aerogen® Pro nebuliser was connected to the NGI induction port using its associated T-piece component and a mouthpiece adapter. Samples (1.35 mL volume) were loaded into the nebuliser reservoir and delivered using a flow rate of 15 L/min. The time taken for dose delivery was 3–4 min. Post nebulisation, the nebuliser, T-piece, throat and stages 1 – 8 of the NGI were thoroughly rinsed with RNase free water to collect the siRNA nanocarriers. The mass of siRNA deposited in each stage was quantified using the Quant-iT™ RiboGreen RNA quantification assay (Invitrogen™, Bleiswijk, NL) and siRNA standard curve (0–500 ng/mL). Triton™X-100 was added to each well at a final concentration of 1 % v/v and shaken for 3 min to release encapsulated siRNA so that the total siRNA concentration (both free and encapsulated) could be quantified. Fluorescence intensity was measured using an Agilent Biotek plate reader (Agilent Technologies, USA) at excitation and emission wavelengths of 485 and 525 nm, respectively. The mass median aerodynamic diameter (MMAD), Fine Particle Fraction (FPF %), and geometric standard deviation (GSD) were calculated using the Copley Inhaler Testing Data Analysis Software (CITDAS) Version 3.10 Wibu (USP 32/pH. Eur. 6.0) from Copley Scientific Limited (Nottingham, UK).

### 2.6 Size and zeta potential measurement

The Z-average (hydrodynamic diameter), polydispersity index (PDI), and zeta potential were measured by dynamic light scattering (DLS) and electrophoretic light scattering (ELS) using a Zetasizer Ultra instrument (Malvern Instruments, Worcestershire, UK). Each nanocarrier formulation was diluted 10-fold in 1X PBS (pH 7.4) up to a 500 µL volume for size analysis and diluted 10-fold in MilliQ ultrapure water up to a 750 µL volume for zeta potential analysis. ELS was carried out using a measurement angle of 12.78° Zetasizer software settings: polystyrene latex as material (refractive index 1.590, absorption 0.010), water as dispersant (viscosity 0.8872 cP, refractive index 1.330), temperature 25 °C and equilibration time of 60 s.

### 2.7 Cryogenic transmission electron microscopy (Cryo-TEM)

siRNA nanocarrier samples containing non-targeting siRNA and suspended in 0.1X PBS buffer were used for cryogenic transmission electron microscopy (cryo-TEM). Samples (3 µL) were applied to glow-discharged Lacey carbon film on 300 Cooper mesh (Ted Pella 01,895-F) and vitrified in liquid ethane using the FEI Vitrobot Mark IV (Thermo Fisher Scientific) at 4 °C with 95 % humidity, a 3–4 s blot time, and 0 blot force. Grids were imaged with the Glacios 2 electron microscope (ThermoFisher Scientific) operating at 200 kV and equipped with the Falcon 4i Direct Electron Detector at the IN-MOL-CELL, International Institute of Molecular and Cell Biology in Warsaw. Images were recorded in an electron counting mode with a physical pixel size of 1.5 Å (nominal magnification of 92,000x) and a total dose of 11.78 e/A^2^.

### 2.8 siRNA encapsulation efficiency

The siRNA encapsulation efficiency (EE %) of each formulation was determined using the Quant-iT™ RiboGreen RNA quantification assay (Invitrogen™, Bleiswijk, NL). 100 µL of sample diluted in Tris-EDTA (TE) buffer was added in triplicate to black 96-well plates (Thermo Fisher Scientific, Dublin, Ireland). The concentration of unencapsulated free siRNA was quantified using either the low-range (0–50 ng/mL) or high-range (0–1 µg/mL) standard curves depending on the concentration in each formulation. The Quanti-iT™RiboGreen RNA reagent was diluted 2000-fold for the low-range standard curve and 200-fold for the high-range standard curve as per the Invitrogen™ protocol. Quanti-iT™RiboGreen RNA reagent was added to the samples, and they were incubated for 5 min at room temperature prior to quantification of free siRNA. To quantify the total RNA (free and encapsulated) in the samples, Triton™ X-100 was added to each well at a final concentration of 1 % v/v and samples were shaken for 3 min to release the encapsulated siRNA. Total siRNA concentration was determined using the high-range standard curve. The fluorescence intensity of the samples post addition of the RiboGreen RNA reagent was measured using a Spark® microplate multimode reader (Tecan, UK) at excitation and emission wavelengths of 485 and 535 nm, respectively. Eq. (1) was used to determine the EE % as follows:

(1) $$siRNA\;EE=100\%\;x\;\frac{\left[total\;RNA\right]-\left[free\;RNA\right]}{\left[total\;RNA\right]}$$

### 2.9 Gel electrophoresis analysis of siRNA binding efficiency

Native polyacrylamide gel electrophoresis (PAGE) was performed to assess the impact of nebulisation on siRNA binding efficiency to its nanocarrier formulations using the Mini-Protean® Tetra system (BioRad, Hercules, *CA*, USA). A 20 % polyacrylamide gel was prepared by mixing 30 % acrylamide-bisacrylamide solution, deionized water, 5X Tris Borate-EDTA (TBE) buffer together followed by addition of ammonium persulfate 10 % w/v and N,N,N′,N′-Tetramethyl-ethylenediamine to initiate polymerization. Following solidification, the gel was placed in an electrophoresis tank and samples were added to the wells. Before loading, the required volume of each sample corresponding to 15 pmol of siRNA was combined in a 2:1 ratio with a loading buffer consisting of bromophenol blue (tracking dye) and glycerol (to increase density). An equivalent amount of naked siRNA (15 pmol) was used as a positive control. In addition, a second sample of each formulation was treated with Triton™ X-100 added to the wells at a final concentration of 3.33 % v/v to dissociate siRNA. RNAse free water was added to the samples accordingly prior to loading to standardise sample volume across all formulations. Samples were electrophoresed at 80 V for 120 min in 1X TBE running buffer. Following this, the gel was stained by incubation in SYBR™ Gold Nucleic Acid Gel Stain (Thermo Fisher Scientific, Dublin, Ireland) for 30 min. Visualisation was carried out using an iBright Imaging System (Thermo Fisher Scientific, Dublin, Ireland).

### 2.10 Cell culture

*In vitro* cell culture experiments were carried out using A549 cells expressing the firefly luciferase gene (luc2) and were obtained from ATCC (Manassas, VA, USA). A549-Luc2 cells were cultured in complete growth media (CGM), which contained Dulbecco’s Modified Eagle Medium (DMEM) supplemented with foetal bovine serum (FBS) 10 %, penicillin-streptomycin 1 % and blasticidin (8 µg/mL) (Thermo Fisher Scientific, Dublin, Ireland) and were maintained as a monolayer culture at 37 °C in 5 % CO_2_.

### 2.11 Cytotoxicity and quantification of luciferase knockdown after siRNA transfection

Cytotoxicity and Fluc knockdown were measured in a multiplexed manner using the CellTiter-Fluor™ cell viability and ONE-Glo™ Luciferase assays (Promega, Madison, WI, USA) and a Spark® microplate multimode reader (Tecan, UK). A549-Luc2 cells were seeded on white 96-well plates (10^4^ cells per well) (Thermo Fisher Scientific, Dublin, Ireland) in CGM and incubated for 24 h to allow them to adhere. After 24 h, the media was replaced with reduced serum media (2 % FBS). Non-nebulised and nebulised samples of siRNA nanocarriers were added to the wells in triplicate at doses of 5 and 10 pmol/well. Naked Fluc siRNA was added as a negative control. Complexes of Fluc siRNA and the commercial transfection reagent, Lipofectamine™ RNAiMAX (Thermo Fisher Scientific, Dublin, Ireland) were also prepared and added to the wells at a dose of 5 pmol/well. Further, Fluc knockdown studies using the LNP formulation were also carried out in the dose range of 0.1 – 2.5 pmol. Cells were incubated with siRNA samples to allow transfection over an 18 h period. Following this, the media containing siRNA nanocarriers was replaced with fresh CGM and the cells were incubated for a further 24 h. Immediately prior to addition of assay reagents, CGM was replaced with DMEM in all wells. CellTiter-Fluor™ reagent (5x concentration) was added to the wells at a volume of 20 µL. The cells were incubated at 37 °C for 40 min and fluorescence was then measured at excitation and emission wavelengths of 380 and 535 nm, respectively. Untreated cells were also seeded as a reference to represent 100 % viability and the % viabilities of each formulation were then determined based upon this. Luciferase activity of the cells was then determined using the ONE-Glo™ Luciferase assay by measuring the emitted luminescence according to the manufacturer’s protocol (Promega, Madison, WI, USA). Fluc knockdown values were normalised to cell viability to ensure any reduction in luminescence recorded was solely due to siRNA-mediated gene silencing and not cell death.

### 2.12 Cellular uptake of nebulised siRNA nanocarriers

Cellular uptake of nebulised siRNA nanocarriers was evaluated using Cy5-siRNA and fluorescence imaging. A549-Luc2 cells were seeded on black glass-bottom 96-well plates (10^4^ cells per well) (ibidi GmbH, Gräfelfing, Germany) in CGM and incubated for 24 h. Samples of nebulised nanocarriers containing Cy5-siRNA were added to the wells at a dose of 5 pmol and were incubated for 6 h. After incubation, the nanocarriers were removed and the cells were washed with Dulbecco’s PBS. The cells were then fixed in 2 % v/v formaldehyde (Thermo Fisher Scientific, Dublin, Ireland) for 15 min and the nuclei stained with DAPI 0.4 µg/mL for 15 min. Fluorescence was then visualised using an EVOS M5000 microscope imaging system (Thermo Fisher Scientific, Waltham, MA, USA) with Cy5 and DAPI channels at 20x.

### 2.13 Statistical analysis

Statistical analysis was performed using GraphPad Prism version 8.4.3. All results represent mean ± SD of three independent experiments (*n* = 3) unless otherwise stated. Statistical significance was determined using one-way, two-way or three-way analysis of variance (ANOVA) depending on the experiment. Post-hoc analysis using Tukey’s or Dunnett’s multiple comparison was also performed. P values < 0.05 were deemed to be statistically significant in all cases.

## 3 Results

### 3.1 Nebulised aerosol droplet size analysis

The Malvern Spraytec laser diffraction system was used to evaluate the aerodynamic properties of the nebulised siRNA nanocarrier formulations. The size distribution of the generated aerosol droplets was analysed, and the following outputs, Volumetric Mean Diameter (VMD), Fine Particle Fraction (FPF %) and flow rate (mL/min) (Table 2) were recorded. The VMD for all siRNA formulations were in the ranges of 4.80 to 4.89 µm and 3.56 to 3.59 µm for the High and Low VMD devices, respectively. A cut-off of 4.95 µm was used to determine the FPF % (< 4.95 µm). The FPF was > 50 % for all formulations, irrespective of nebuliser device used, indicating a large fraction of the emitted droplets have potential for peripheral lung deposition. Notably, the FPF was higher for formulations aerosolised using the low VMD device.

**Table 2:** Output characteristics of siRNA nanocarrier formulations when nebulised with the High and Low VMD devices. Analysis carried out using laser diffraction. Results represent mean ± SD of 3 independent nebulisation runs (*n* = 3).

High VMD Device Output Characteristics			
Formulation	VMD (µm)	FPF ( %) < 4.95 µm	Flow rate (mL/min)
PBS	4.83 ± 0.08	51.00	0.5 ± 0.04
non-PEG LPXs	4.89 ± 0.07	50.56	0.46 ± 0.04
PEG LPXs	4.80 ± 0.05	51.47	0.47 ± 0.04
LNPs	4.89 ± 0.08	50.59	0.47 ± 0.04

Low VMD Device Output Characteristics			
Formulation	VMD (µm)	FPF ( %) < 4.95 µm	Flow rate (mL/min)
PBS	3.34 ± 0.06	69.71	0.25 ± 0.01
non-PEG LPXs	3.59 ± 0.08	65.53	0.32 ± 0.01
PEG LPXs	3.58 ± 0.01	66.42	0.28 ± 0.01
LNPs	3.56	66.12	0.31 ± 0.02

### 3.2 Cascade impaction using a next generation impactor (NGI)

The aerodynamic mass deposition profile of each siRNA nanocarrier formulation nebulised using the High VMD device was assessed via an NGI cascade impaction apparatus. Across all formulations, the mean MMAD, FPF ( % < 5 µm) and GSD values were in the ranges of 4.03 - 4.84 µm, 45.98 – 59.40 % and 2.19 – 2.46, respectively (Table 3). The mass distribution profiles across the respective stages of the NGI for each formulation are provided in the Supplementary Data, (**Fig S2-S4**). The average mass balance across formulations was 61.18 – 79.56 %.

**Table 3:** Aerodynamic particle size distribution results for siRNA nanocarrier formulations when nebulised with the High VMD device. Results represent mean ± SD of 3 independent nebulisation runs (*n* = 3).

Formulation	MMAD (µm)	FPF (%) < 5 µm	GSD
non-PEG LPXs	4.84 ± 0.56	45.98 ± 3.58	2.46 ± 0.12
PEG LPXs	4.12 ± 0.67	56.21 ± 4.18	2.26 ± 0.16
LNPs	4.03 ± 0.93	59.40 ± 10.36	2.19 ± 0.27

### 3.3 Size and zeta potential of siRNA nanocarriers

**Fig. 1:**
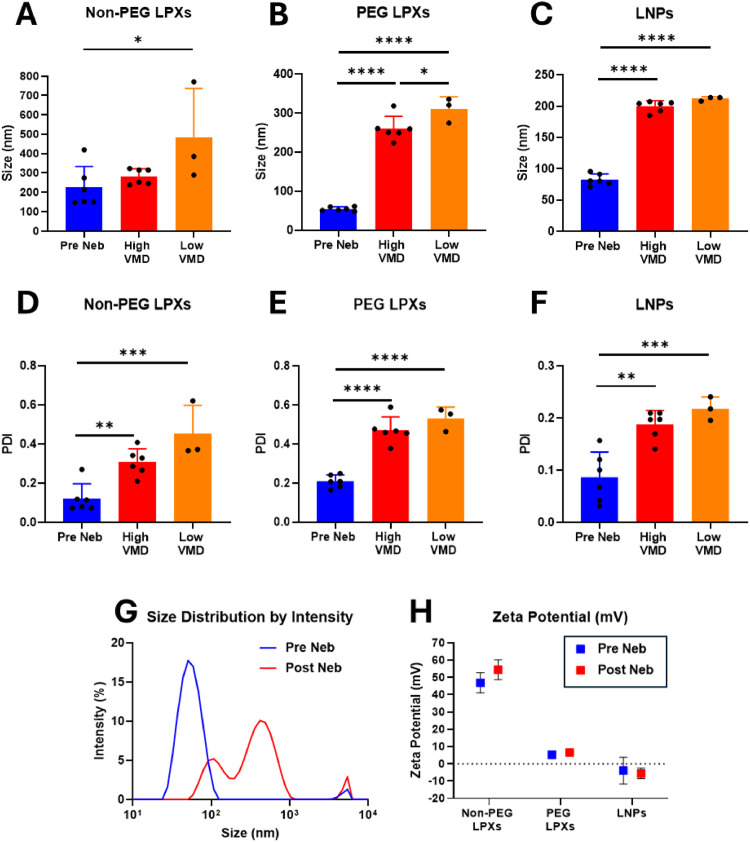
Physicochemical characterisation of siRNA nanocarriers pre and post nebulisation; (A-C) size, (D-F) PDI, (G) size intensity distribution of PEG LPXs pre and post nebulisation with High VMD Device and (H) zeta potential results. Black dots in A-F represent independent experiments. Data represents mean ± SD of 3–6 independent experiments (*n* = 3- 6). * p values < 0.05, ** p values < 0.01, *** p values < 0.001 and **** p values <0.0001.

DLS analysis was carried out to measure the size of the different siRNA nanocarriers both prior to and post nebulisation. The non-PEG LPXs had the largest size pre nebulisation ∼ 226 nm, while all other formulations were considerably smaller in size < 85 nm (Fig. 1**A-C**)**.** The PDI of all formulations prior to nebulisation was low (≤ 0.2) (Fig. 1**D-F**). Nebulisation with both devices (High and Low VMD) caused a statistically significant increase in size for all formulations except for the non-PEG LPXs (Low VMD only) (Fig. 1**A**). In some samples the size distributions were described by more than one population, for example the PEG LPXs both pre and post nebulisation (Fig. 1**G**). Where ≥ 2 size populations are present, z-average diameter and PDI become less accurate, and the intensity distribution is a more reliable representation of the sample. Nevertheless, z-average and PDI remain useful for comparison purposes between different samples (Panalytical, 2023). Representative size intensity distribution spectra of the three siRNA nanocarriers are provided in the Supplementary Data, (**Fig S5-S7**). Several of the nebulised, non-PEG LPXs samples contained a larger-sized population that was greater than the 10 µm operational limit of the zetasizer equipment possibly indicating aggregation post nebulisation. This observation is reflected in the increased PDI for the non-PEG LPXs post nebulisation (Fig. 1**D**). Potential aggregation highlighted by a larger size population was also present for several of the nebulised PEG LPXs samples (Fig. 1**B**). PDI was found to increase significantly across all formulations post nebulisation (Fig. 1**D-F**). Regarding the impact of VMD, the PEG LPXs were the only formulation to exhibit a statistically significant difference in size between the samples nebulised using the High and Low VMD devices (Fig. 1**B**). The non-PEG LPXs had the highest zeta potential of all formulations (+ 46.9 mV pre nebulisation), while all other formulations exhibited zeta potential values close to neutral, 0 mV (Fig. 1**H**)**.** Nebulisation did not result in a statistically significant change in zeta potential across any of the three nanocarrier types.

### 3.4 Cryogenic transmission electron microscopy (Cryo-TEM)

**Fig. 2:**
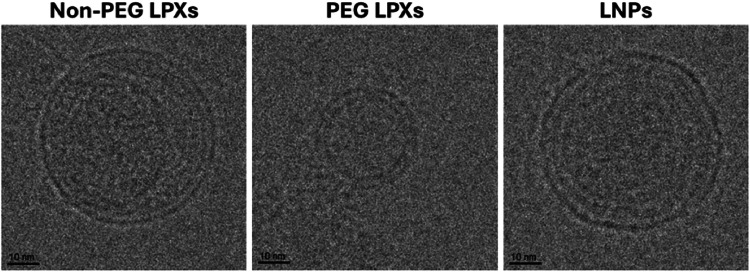
Representative cryo-TEM images of siRNA nanocarriers (pre nebulisation), scale bars represent 10 nm.

Cryo-TEM analysis was performed to provide an insight into siRNA nanocarrier morphology and size. All three nanocarrier formulations had spherical morphologies with lamellar structures (Fig. 2). The PEG LPXs exhibited a unilamellar arrangement while the non-PEG LPXs and LNPs both appeared multilamellar. The approximate particle diameters of the non-PEG LPXs, PEG LPXs and LNPs based on cryo-TEM imaging were 55 nm, 30 nm and 60 nm, respectively, which were smaller than corresponding mean sizes determined using DLS analysis, particularly for the non-PEG LPXs. However, cryo-TEM does not account for the particle’s hydration layer, which is captured in DLS measurements (Webb et al., 2020). Further, cryo-TEM measurements necessitated the use of PBS buffer at a lower (0.1X PBS) concentration. It has been shown that increasing buffer strength can increase liposome size, in particular for cationic liposomes (Jia et al., 2025; Lou et al., 2019). To confirm this, DLS analysis was carried out on siRNA nanocarriers formulated in 0.1X PBS (concentration used for cryo-TEM). Results confirmed that non-PEG LPXs size were significantly smaller (approximately 80 nm) compared to those prepared at 1X PBS concentration (**Fig S9**).

### 3.5 siRNA encapsulation efficiency and gel electrophoresis analysis

**Fig. 3:**
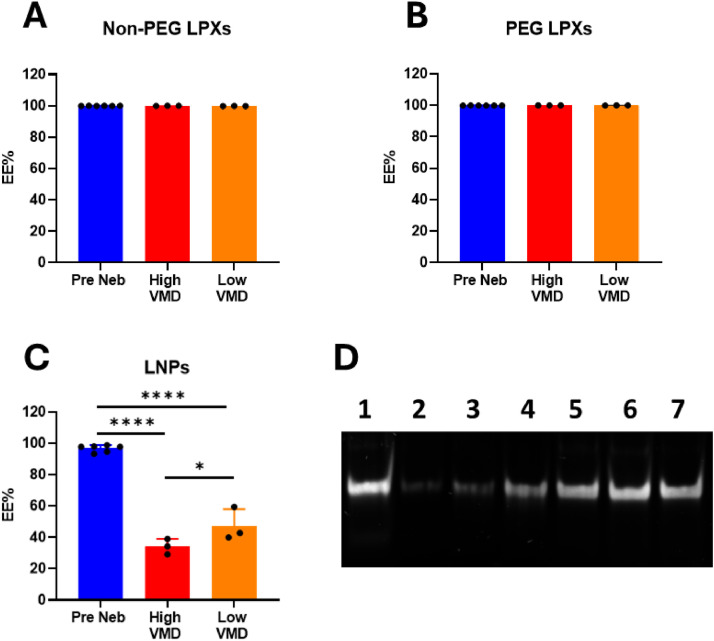
A) Encapsulation efficiency (EE %) pre and post nebulisation of (A) Non-PEG LPXs, (B) PEG LPXs and (C) LNPs. Black dots in graph represent independent experiments. Data represents mean ± SD of 3–6 independent experiments (*n* = 3–6). * p values < 0.05 and **** p values < 0.0001. (D) Gel electrophoresis of nebulised and Triton™ X-100 treated siRNA nanocarriers. Nebulised samples are shown in lanes 2–4 and Triton™ X-100 treated nebulised samples are shown in lanes 5–7. Lane 1: Naked siRNA control. Lane 2: Non-PEG LPXs. Lane 3: PEG LPXs. Lane 4: LNPs. Lane 5: Non-PEG LPXs + Triton™ X-100. Lane 6: PEG LPXs + Triton™ X-100. Lane 7: LNPs + Triton™ X-100.

The EE % of siRNA in all formulations was high pre nebulisation (> 96 %) as per RiboGreen analysis (Fig. 3**A-C**). The EE % of the LNPs was significantly reduced post nebulisation with both nebuliser devices to 34.13 % (High VMD) and 47.36 % (Low VMD). The EE % of the LNPs samples nebulised with the Low VMD device was found to be statistically higher than those nebulised using the High VMD device (Fig. 3**C**). The EE % of the LPX formulations remained unchanged post nebulisation irrespective of nebuliser device used (Fig. 3**A-B**). Gel electrophoresis was performed to assess siRNA binding efficiency and integrity in each nanocarrier post nebulisation. In the case of LNPs, a free siRNA band was clearly visible indicating a sizeable amount of unbound siRNA, which is consistent with the RiboGreen results. Further, Triton™ X-100 was added to dissociate the siRNA from the nebulised nanocarriers. A clear band of free siRNA comparable to the siRNA control was observed in each lane post Triton™ X-100 addition confirming that all formulations had maintained siRNA integrity during nebulisation (Fig. 3**D**)**.**

### 3.6 Cytotoxicity of siRNA nanocarriers

**Fig. 4:**
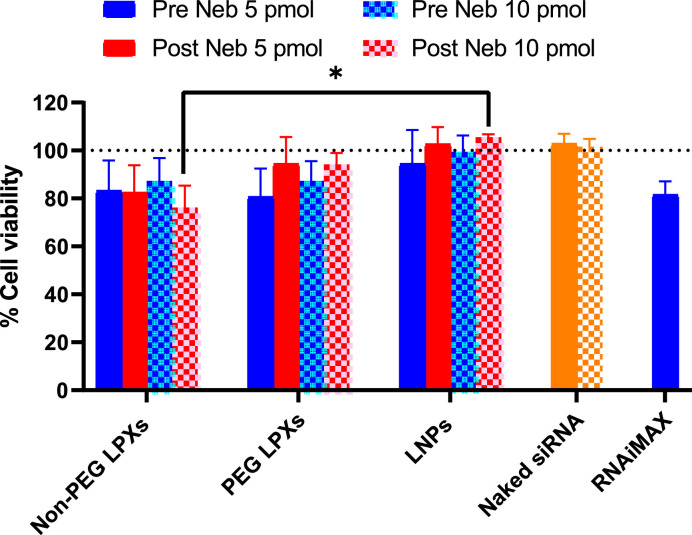
Cell viability of siRNA nanocarriers pre and nebulisation. Results represent mean ± SD of 3 independent experiments (*n* = 3). *p value <0.05. Dashed line represents 100 % viability. Naked siRNA at 5 pmol (solid bar) and 10 pmol (checkered) doses and RNAiMAX (5 pmol dose) also shown.

The CellTiter-Fluor™ Cell Viability Assay was used to evaluate the cytotoxicity of the nanocarrier formulations in A549 cells. Nanocarrier samples were analysed pre and post nebulisation to determine the potential impact of nebulisation on cytotoxicity. Each nanocarrier was evaluated at two doses: 5 pmol and a higher dose of 10 pmol to account for the anticipated varying levels of dosage requirement across the different siRNA nanocarrier types. Naked Fluc siRNA and Lipofectamine™ RNAiMAX (5 pmol only) were used as negative and positive controls, respectively in the Fluc knockdown assay. The non-PEG LPXs post nebulisation (10 pmol dose) had the lowest cell viability value (76.3 %). All other formulations showed cell viability values > 80 % indicating low cytotoxicity (Fig. 4). It was shown that nebulisation did not cause any significant changes in the cell viability of any of the nanocarrier formulations investigated in this study.

### 3.7 siRNA luciferase knockdown studies

**Fig. 5:**
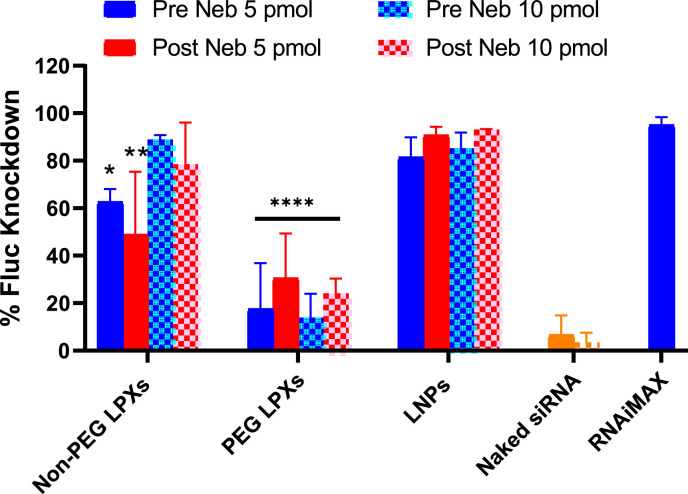
Luciferase knockdown of siRNA nanocarriers pre and post nebulisation. Results represent mean ± SD of 3 independent experiments (*n* = 3). Naked siRNA at 5 pmol (solid bar) and 10 pmol (checkered) doses and RNAiMAX (5 pmol dose) also shown. *p values <0.05, **p values <0.01 and ****p values <0.0001 vs RNAiMAX control.

Transfection was carried out at two different doses: 5 and 10 pmol/well. Knockdown values of each formulation were determined relative to untreated cells (100 % Fluc expression) (Fig. 5). Considerable variability in the data was observed for the non-PEG LPXs post nebulisation. The PEG LPXs data was also variable and corresponded to the lowest transfection efficiency with a maximum of 30 % knockdown observed post nebulisation. Knockdown was high in the case of LNPs and was comparable to Lipofectamine™ RNAiMAX. LNP-mediated knockdown did not decrease after nebulisation (93 % post nebulisation at a 10 pmol dose). The Fluc knockdown in the naked siRNA control wells was expectedly low (< 10 %). Overall, nebulisation did not significantly affect Fluc knockdown for any formulation. The range in Fluc knockdown values observed was due to differences in formulation type and also dose in the case of the non-PEG LPXs.

**Fig. 6:**
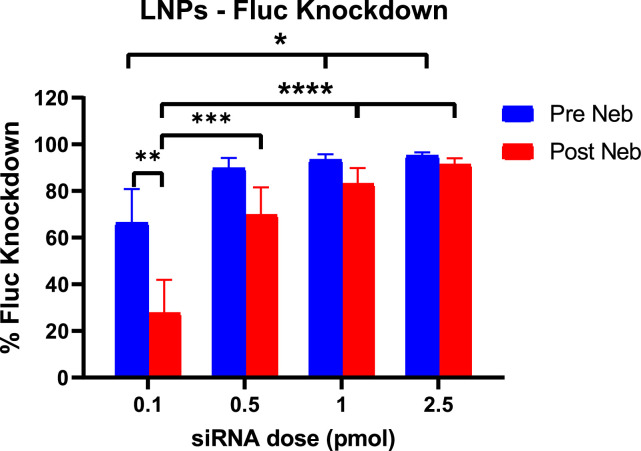
Luciferase knockdown of siRNA-LNPs pre and post nebulisation. Results represent mean ± SD of 3 independent experiments (*n* = 3). *p values <0.05, **p values <0.01 *** p values <0.001 and ****p values <0.0001.

Considering the EE % and comparable Fluc knockdown for the siRNA-LNP samples pre and post nebulisation, further transfection experiments were performed at lower doses in the range 0.1 – 2.5 pmol. Fluc knockdown was significantly lower post nebulisation (27.6 %) than pre nebulisation (66.4 %) when siRNA-LNPs were administered at the lowest dose (0.1 pmol). In the case of post-nebulisation samples, Fluc knockdown was significantly higher at all doses compared to 0.1 pmol (Fig. 6).

### 3.8 Cellular uptake of nebulised siRNA nanocarriers

**Fig. 7:**
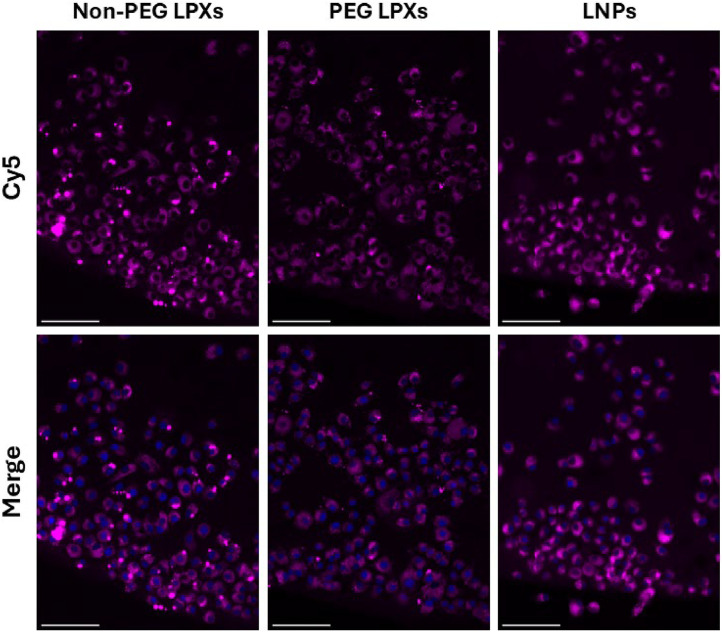
Fluorescence images of A549-Luc2 cells following 6 h incubation with nebulised nanocarriers containing Cy5-siRNA (pink). Nuclei (blue) are stained with DAPI. Scale bars = 100 µm.

Fluorescence imaging with Cy5-siRNA was used to evaluate cellular uptake of the siRNA nanocarriers post nebulisation. A Cy5 signal is visible for all three nanocarrier formulations signifying the uptake of Cy5-siRNA nanocarriers into cells (Fig. 7). The Cy5 signal intensity was lowest for the PEG LPX treated cells implying that intracellular uptake of PEG LPXs was lower than that of the other formulations.

## 4 Discussion

This study sought to investigate the interplay between two important aspects of nebulised siRNA delivery, namely 1) the size of the aerosol droplet produced by the device and 2) the physicochemical properties and stability of a range of siRNA nanocarrier formulations. The VMD results for all siRNA formulations produced using two Aerogen® Pro vibrating mesh nebulisers were in a narrow range for both the High (4.80 to 4.89 µm) and Low (3.56 to 3.59 µm) VMD devices. Therefore, the laser diffraction data demonstrates uniform droplet size can be achieved in the case of multiple lipid-based nanocarrier types including lipoplexes (PEGylated and non-PEGylated) and LNPs, irrespective of whether a Low or High VMD device is used. Cascade impaction was also used to assess aerodynamic performance. In the case of the mass deposition data, a greater range in MMAD values across formulations (4.03 - 4.84 µm) was observed. However, all values were < 5 µm indicating potential to deposit siRNA in the lower lung regions (Pleasants and Hess, 2018).

Regarding the influence of the nebulisation process on the physicochemical stability of siRNA nanocarriers, it was found that altering aerosol droplet size profile via changing Aerogen® Pro vibrating mesh nebuliser did not result in any significant differences in observed nanocarrier stability. Both devices, High and Low VMD, produced comparable results. Particle aggregation was apparent in the case of both lipoplex formulations and siRNA EE % was significantly reduced in the LNPs. Previous studies reported the positive influence of PEG increasing LNP robustness during nebulisation (Kim et al., 2022; Lokugamage et al., 2021). However, in this study, incorporation of PEG in the lipoplexes did not appear to stabilise them during nebulisation and prevent aggregation. Regarding *in vitro* performance in A549 cells, cell viability and Fluc knockdown were found to be mostly impacted by formulation choice. The C12–200 LNP formulation, which has not previously been assessed for vibrating mesh nebuliser-mediated siRNA delivery, shows the best potential with high transfection efficiency despite the drop in EE % post nebulisation. However, optimisation is required to improve its siRNA EE % post nebulisation for example, increasing the selected mass ratio of C12–200: siRNA (5:1).

The Aerogen® Pro nebuliser contains a perforated mesh, which when vibrated causes the solution to be pumped though the mesh apertures in the form of aerosol droplets (Ghazanfari et al., 2007). This high energy input process can exert shear stress on nanoparticles, albeit to a considerably lesser degree than the jet and ultrasonic nebuliser designs (Hertel et al., 2014). Additionally, nebulisation results in temporary nanoparticle exposure to an air-liquid interface that can also cause instability in liposomes (Lehofer et al., 2014). Both LPX formulations contained 45 – 50 mol % cholesterol as a stability enhancer to increase the rigidity of their lipid bilayers (Briuglia et al., 2015). Despite this, both LPX formulations displayed instability during nebulisation culminating in nanocarrier aggregation and this occurred for both High and Low VMD devices. DMPE-PEG_2000_ was chosen as a LPX component with the aim of enhancing liposome stability (Jaradat et al., 2023) and robustness during vibrating mesh nebulisation. However, PEG is also known to become unstable when exposed to external stresses including heat and mechanical forces (Pasarin et al., 2023). It is possible that a percentage of the PEG molecules may have been lost from the surface of the PEG LPXs during nebulisation causing aggregation (Cai et al., 2011). Szabova et al. also reported an increase in the number of larger PEGylated liposomes particles after nebulising with a vibrating mesh nebuliser (Szabová et al., 2021). The authors observed that immobilisation of water molecules by hydrophilic PEG caused increased rigidity of the liposome’s lipid bilayer (Szabová et al., 2021). Since a partial loss of PEG during nebulisation could reduce lipid bilayer rigidity, this may possibly explain the instability in our PEG LPXs.

The LNPs underwent comparable increases in size and PDI when nebulised with the High VMD (200 nm, 0.188) and Low VMD (212 nm, 0.218) devices. Despite the increases in particle size, LNPs remained of an appropriately small size for endocytosis into cells and had an acceptably low PDI (<0.3) (Danaei et al., 2018), which is important for consistent therapeutic efficacy (Robertson et al., 2016). The increase in LNP size post nebulisation is consistent with other studies involving nebulised LNPs (Kim et al., 2020; Zhang et al., 2020). Interestingly, Jiang et al. noted that mRNA LNP size increases during nebulisation were reduced when PBS buffer, also used to suspend our LNPs, was replaced with sodium acetate and a branched PEG excipient, which they attributed to an increased resistance to nebulisation-induced aggregation (Jiang et al., 2023).

The high EE % of both lipoplex formulations that was unaltered post nebulisation, irrespective of vibrating mesh nebuliser device used herein, contrasts with several other studies involving nebulised liposomes (Elhissi et al., 2012; Kleemann, 2006; Klein et al., 2021). For instance, in a study by Kleemann et al. the percentage encapsulation of the cargo, 5(6)-carboxyfluorescein within DPPC-based liposomes dropped to as low as 39 % following nebulisation (Kleemann, 2006). In that study, the authors used the neutral lipid, DPPC (Kleemann, 2006), which could explain loss of the cargo during nebulisation. In this study, the high EE % in both LPX formulations post nebulisation was unexpected given their size increase indicating destabilisation. However, it is conceivable that siRNA is securely bound within the LPXs throughout nebulisation due to electrostatic interaction with DOTAP. Furthermore, DOTAP’s positive charge could also enable prompt complexation of any unbound siRNA lost during nebulisation with the lipoplexes. Additionally, a high N/P ratio of 5:1 was used in our LPX formulations to guarantee sufficient DOTAP molecules to electrostatically bind all available siRNA during manufacture and minimize the quantity of residual, free siRNA (Hattori et al., 2015).

The EE % of the LNPs nebulised using both the High VMD and Low VMD devices significantly reduced to < 50 %. It is likely that the forces exerted by the nebuliser’s vibrating mesh impacted the physical integrity of the LNPs resulting in leakage of siRNA. This finding was supported by the gel electrophoresis results which showed a clear band of migrating siRNA present in the well containing the nebulised LNPs. Other recent studies have also noted a substantial decrease in RNA EE % in LNPs following nebulisation (Kim et al., 2022; Miao et al., 2023). Kim et al. observed a decrease in mRNA EE % to <40 % in all nebulised LNP compositions investigated, which they attributed to the rearrangement of the LNP structure during nebulisation (Kim et al., 2022). However, Kim et al. used a different ionizable lipid, Dlin-MC3-DMA (Kim et al., 2022) to our own study and it is known that choice and molar % of lipid components heavily influence final LNP structural arrangement (Leung et al., 2015). LNPs in general, comprise an electron dense core of inverted micelles consisting of ionizable lipid encircling siRNA molecules (Leung et al., 2015; Mendonça et al., 2023). Unlike, the permanently cationic DOTAP present in the LPXs, C12–200 is neutral at pH 7.4 (Miao et al., 2021) therefore it may have less electrostatic interaction with siRNA, and consequently a reduced impact on mitigating siRNA egress during nebulisation. Notably, in a recent study, Slaughter et al. observed that siRNA LNP EE % could be maintained during nebulisation by reducing the pH of their citrate buffer medium from pH 6.0 to 4.0 which they attributed to Dlin-MC3-DMA, the ionizable lipid employed, acquiring a positive charge (Slaughter et al., 2025).

All formulations had low cytotoxicity indicated by high % viability > 80 % except for the nebulised non-PEG LPXs at the higher 10 pmol dose, which had a marginally lower value of 76 %. The slightly reduced cell viability of the non-PEG LPXs can be attributed to its high cationic charge known to have undesirable toxic effects (Rietwyk and Peer, 2017). Although, the high N/P ratio of 5:1 is advantageous for producing lipoplexes with high siRNA encapsulation post aerosolization, this represents a potential trade-off in formulation optimisation as a reduced cationic lipid mass relative to RNA is desirable to minimise cytotoxicity. Moreover, cationic nanocarriers can electrostatically bind to anionic mucin proteins present in the lungs’ mucus leading to low mucopenetration (Lai et al., 2009) and ultimately impaired intracellular siRNA delivery. In addition to their potential toxicity, poorer mucopenetration is another challenge to be considered when administering highly cationic nanocarriers into the lungs.

Nebulisation did not significantly affect the cytotoxicity of any of the formulations investigated. The % Fluc knockdown levels varied significantly reflecting the diverse array of nanocarriers assessed and their contrasting transfection efficiency *in vitro*. The PEG LPXs had the poorest transfection capability overall. Notably, PEGylation is known to impede both cellular uptake and endosomal escape of nanoparticles due to steric hindrance of the interaction with the cellular and endosomal membranes, a prerequisite for cytosolic RNA delivery (Li et al., 2014). Fluorescence imaging results suggest that poor PEG LPX-mediated knockdown may be partially due to low intracellular delivery. The low gene knockdown observed may also result from inefficiency in escaping the endosome, however elucidation of such intracellular activity is beyond this current study’s scope. In contrast, despite a substantial drop in EE %, the LNPs still produced effective % Fluc silencing post nebulisation at the 5 pmol dose. Owing to their efficient design, LNPs have been shown to deliver a greater proportion of their RNA payload into target cells compared to other vector types including lipoplexes (Kubota et al., 2017). Hence, additional Fluc knockdown experiments were performed at doses ≤ 2.5 pmol/well to further elucidate the impact of nebulisation on LNP-mediated transfection. Fluc knockdown was significantly reduced post nebulisation at a 0.1 pmol dose highlighting the effect of lower EE % at this dose.

It should be noted that the *in vitro* testing in this study was limited to A549 alveolar epithelial cells. *In vitro* models of greater complexity such as cell co-cultures with air-liquid interfaces (D’Angelo et al., 2018) may more accurately project *in vivo* performance. However, such work was considered out of scope in our studies that primarily focused on aerodynamic properties and siRNA nanocarrier physicochemical characteristics and could be the subject of future investigations.

## 5 Conclusion

Our study has shown that the Aerogen® Pro vibrating mesh nebuliser could produce aerosols of an appropriate droplet size for siRNA inhalation, and remained consistent across distinct nanocarrier types, in this case, lipoplexes and LNPs. Using two nebulisers which generated different aerosol size distributions did not result in any discernible changes in the physical properties of nebulised siRNA nanocarriers. The High and Low VMD devices both had a similar impact on nanocarrier stability resulting in aggregation of both lipoplex types and significant loss of siRNA cargo in the LNPs. PEGylation did not enhance the nebulisation stability of the DOTAP:cholesterol lipoplexes herein thus formulation optimisation is required. The influence of siRNA formulation rather than nebulisation with the Aerogen® Pro nebuliser was found to be more impactful towards *in vitro* performance with varying levels of Fluc knockdown observed. The C12–200 based LNPs elicited high Fluc knockdown in A549 cells and the formulation could be further optimised in future studies to improve its siRNA encapsulation post nebulisation. Overall, our results show that the Aerogen® Pro nebuliser offers the potential to achieve peripheral lung deposition of siRNA using different delivery vector types. However, the impact of vibrating mesh nebulisation on the physical properties of siRNA nanocarriers can vary considerably, therefore nanocarrier formulation and its optimisation are important to maximise siRNA delivery and efficacy in the case of nebulised delivery to the lung.

## CRediT authorship contribution statement

**Michael T. Neary:** Writing – review & editing, Writing – original draft, Methodology, Investigation, Formal analysis, Data curation, Conceptualization. **Lianne M. Mulder:** Writing – review & editing, Methodology. **Ciaran O. Leime:** Writing – review & editing, Writing – original draft, Methodology, Investigation, Formal analysis. **Ronan MacLoughlin:** Writing – review & editing, Methodology. **Brunella Grassiri:** Methodology, Investigation, Formal analysis. **Łukasz Baranowski:** Writing – original draft, Methodology, Investigation, Formal analysis. **Piotr S. Kowalski:** Writing – review & editing, Methodology. **Abina M. Crean:** Writing – review & editing, Writing – original draft. **Katie B. Ryan:** Writing – review & editing, Writing – original draft, Supervision, Resources, Project administration, Funding acquisition, Formal analysis, Data curation, Conceptualization.

## Declaration of competing interest

Ronan Mac Loughlin and Ciaran Leime are employees of Aerogen Limited. Dr. Ronan MacLoughlin has acted as an editor of the Special Issue, Pulmonary Drug Delivery 2 in European Journal of Pharmaceutical Sciences. The remaining authors declare no competing interests.

## Data Availability

Data will be made available on request.

## Appendix Supplementary materials

Image, application 1
