# Supplementary material for: Pulmonary delivery of siRNA lipoplexes and lipid nanoparticles using a vibrating mesh nebuliser
Source: Eur J Pharm Sci. 2026 Jan 1;216:107386. doi: 10.1016/j.ejps.2025.107386 (PMC12700850; doi:10.1016/j.ejps.2025.107386)
Supplement: Supplementary file 1 [file mmc1.docx]

**Pulmonary Delivery of siRNA Lipoplexes and Lipid Nanoparticles using a Vibrating Mesh Nebuliser**

Michael T. Neary^a,b^, Lianne M. Mulder^b^, Ciaran O. Leime^c^, Ronan MacLoughlin^c^, Brunella Grassiri^d^, Łukasz Baranowski^e^ Piotr S. Kowalski^b,f^, Abina M. Crean^a,b^, Katie B. Ryan^a,b*^

^a^ SSPC, the SFI Research Centre for Pharmaceuticals, School of Pharmacy, University College Cork, Ireland

^b^ School of Pharmacy, University College Cork, Ireland

^c^ Aerogen Ltd. Galway Business Park, Galway, Ireland

^d^ School of Pharmacy and Pharmaceutical Sciences, Panoz Institute, Trinity College Dublin, Dublin 2, Ireland

^e^ Preclinical Drug Development Facility at IN-MOL-CELL, International Institute of Molecular and Cell Biology, Trojdena 4, 02-109 Warsaw, Poland

^f^ APC Microbiome, University College Cork, Ireland

^*^ Corresponding author:

Dr Katie B Ryan

SSPC, the SFI Research Centre for Pharmaceuticals,

School of Pharmacy,

University College Cork,

College Rd., CorkT12 K8AF, Ireland.

Email address: [Katie.Ryan@ucc.ie](mailto:Katie.Ryan@ucc.ie) (Katie B Ryan)

Tel 00353-21-4901680

| **Author** | **ORCID iD** |
| --- | --- |
| Michael T. Neary | 0009-0001-8477-7321 |
| Lianne M. Mulder | 0000-0002-3617-7188 |
| Ciaran O. Leime | 0000-0002-7420-4159 |
| Ronan MacLoughlin | 0000-0002-3164-1607 |
| Brunella Grassiri | 0000-0002-6404-9845 |
| Łukasz Baranowski | 0009-0006-6841-9686 |
| Piotr S. Kowalski | 0000-0001-8607-0189 |
| Abina M. Crean | 0000-0001-6171-0303 |
| Katie B. Ryan | 0000-0002-6236-2977 |


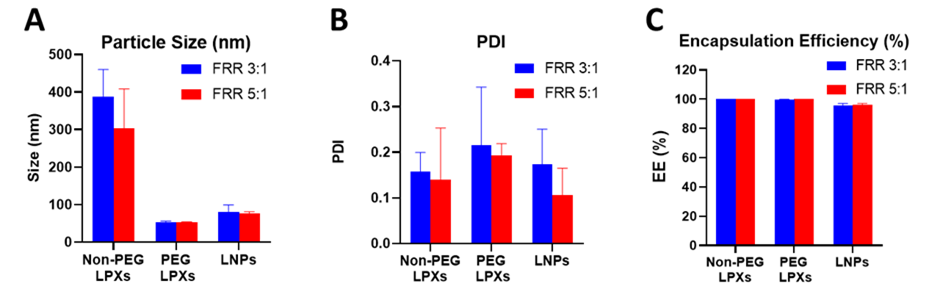


Supplementary Figure 1 Impact of flow rate ratio (FRR) on (A) size, (B) PDI and (C) encapsulation efficiency (EE%). Data represents mean +/- SD of 3 independent experiments (n =3).

Supplementary Figure 2 siRNA mass distribution results for Non-PEG LPXs using the High VMD device for cascade impaction analysis. Results represent mean +/- SD of 3 independent nebulisation runs (n=3).

Supplementary Figure 3 siRNA mass distribution results for PEG LPXs using the High VMD device for cascade impaction analysis. Results represent mean +/- SD of 3 independent nebulisation runs (n=3).

Supplementary Figure 4 siRNA mass distribution results for LNPs using the High VMD device for cascade impaction analysis. Results represent mean +/- SD of 3 independent nebulisation runs (n=3).

Supplementary Figure 5 Size intensity distribution spectra of siRNA nanocarriers pre nebulisation; (A-C) Non-PEG LPXs, (D-F) PEG LPXs and (G-I) LNPs. Spectra represent independent batches for each formulation.

Supplementary Figure 6 Size intensity distribution spectra of siRNA nanocarriers nebulised with High VMD device; (A-C) Non-PEG LPXs, (D-F) PEG LPXs and (G-I) LNPs. Spectra represent independent batches for each formulation.

Supplementary Figure 7 Size intensity distribution spectra of siRNA nanocarriers nebulised with Low VMD device; (A-C) Non-PEG LPXs, (D-F) PEG LPXs and (G-I) LNPs. Spectra represent independent batches for each formulation.


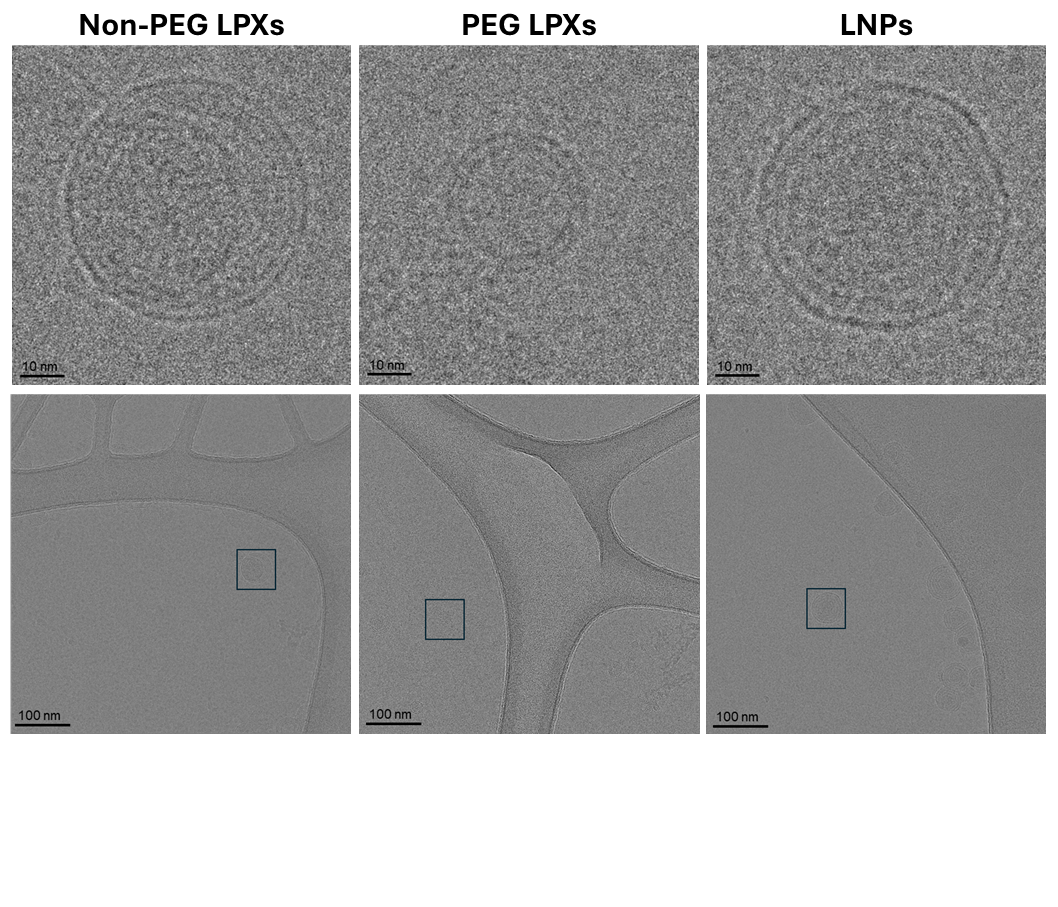


Supplementary Figure 8 Cryo-TEM images of siRNA nanocarriers including lower magnification images (bottom). The boxed regions correspond to the higher magnification images (top). Scale bars represent 10 and 100 nm.

Supplementary Figure 9 DLS size measurements of siRNA nanocarriers formulated in 0.1X PBS. Data represents mean +/- SD (n =3).
